# Earthworms Produce phytochelatins in Response to Arsenic 

**DOI:** 10.1371/journal.pone.0081271

**Published:** 2013-11-22

**Authors:** Manuel Liebeke, Isabel Garcia-Perez, Craig J. Anderson, Alan J. Lawlor, Mark H. Bennett, Ceri A. Morris, Peter Kille, Claus Svendsen, David J. Spurgeon, Jacob G. Bundy

**Affiliations:** 1 Department of Surgery and Cancer, Imperial College London, London, United Kingdom; 2 Centre for Ecology and Hydrology, Wallingford, United Kingdom; 3 School of Biosciences, University of Cardiff, Cardiff, United Kingdom; 4 Centre for Ecology and Hydrology, Lancaster, United Kingdom; 5 Department of Life Sciences, Imperial College London, London, United Kingdom; Queen Mary University of London, United Kingdom

## Abstract

Phytochelatins are small cysteine-rich non-ribosomal peptides that chelate soft metal and metalloid ions, such as cadmium and arsenic. They are widely produced by plants and microbes; phytochelatin synthase genes are also present in animal species from several different phyla, but there is still little known about whether these genes are functional in animals, and if so, whether they are metal-responsive. We analysed phytochelatin production by direct chemical analysis in *Lumbricus rubellus* earthworms exposed to arsenic for a 28 day period, and found that arsenic clearly induced phytochelatin production in a dose-dependent manner. It was necessary to measure the phytochelatin metabolite concentrations directly, as there was no upregulation of phytochelatin synthase gene expression after 28 days: phytochelatin synthesis appears not to be transcriptionally regulated in animals. A further untargetted metabolomic analysis also found changes in metabolites associated with the transsulfuration pathway, which channels sulfur flux from methionine for phytochelatin synthesis. There was no evidence of biological transformation of arsenic (e.g. into methylated species) as a result of laboratory arsenic exposure. Finally, we compared wild populations of earthworms sampled from the field, and found that both arsenic-contaminated and cadmium-contaminated mine site worms had elevated phytochelatin concentrations.

## Introduction

Long-term mining contamination can adversely affect soil invertebrate communities, with consequences for ecosystem services [1,2]. Earthworms form a large portion of the invertebrate community in many soils, and play a key role in soil aeration and processing of organic matter, and are considered to be important environmental sentinel organisms [3]. The species *Lumbricus rubellus* has been widely used in ecotoxicology and, because it is readily found in the field, it is suitable both for laboratory experiments and for studying natural populations. There have been several studies on the effects of potentially toxic elements on natural populations of *L. rubellus*, including arsenic contamination around mine sites [4]. There is clear evidence of genetic adaptation to arsenic in earthworms from former mine sites, with population-level differences in biochemical arsenic speciation [5,6], and also biological responses to arsenic [7]. However, there have been relatively few studies of earthworm responses to arsenic at the molecular level in a controlled laboratory environment, and we still do not understand all of the means by which earthworms detoxify arsenic.

Phytochelatins (PCs) are non-ribosomal peptides, produced from condensation of glutathione molecules by phytochelatin synthase (PCS). They strongly chelate soft metal ions, with cadmium being the canonical example, but can also detoxify arsenic, copper, and mercury. They have the general structure (GluCys)_n_Gly, written as PC_n_, where n ranges from 2 to 11 [8,9]. As the name suggests, they were originally thought to be found only in plants, but two independent studies demonstrated that the PCS from the nematode *C. elegans* produced PCs when cloned into an appropriate microbial host and that a *C. elegans* PCS knockout was hypersensitive to cadmium [10,11]. Treating *C. elegans* with cadmium also led to an increase in tissue concentrations of PC_2_ and PC_3_ [12,13]. More recently, a functional PCS has been demonstrated in a second animal phylum (Platyhelminthes), as the *Schistosoma mansoni* PCS is also cadmium-responsive when cloned into baker’s yeast [14], although PC production has not yet been observed in the S. *mansoni* flukes themselves [15]. 

PCS genes are found in species from several other metazoan phyla, including Annelida, Cnidaria, Echinodermata, Chordata, and Mollusca (both Gastropoda and Bivalvia classes) [16,17]. A BLAST [18] search (against the *C. elegans* PCS) of the NCBI nucleotide and EST databases (as of August 2012) gives an additional hit from another metazoan phylum, Hemichordata (for the acorn worm *Saccoglossus kowalevskii*, NCBI reference sequence XM_002730326), and for another class (Cephalochordata) within Mollusca (for the squid *Doryteuthis pealii*, GenBank ID JK333425.1). Excitingly, PCS expression increases in an earthworm species in response to cadmium [19], implying that PC production may be a means by which earthworms detoxify metal ions, although direct production of PCs has not yet been observed in earthworms. There is no evidence that we are aware of that arsenic can stimulate PC synthesis in metazoans, but PC production in response to arsenic is common in plants [20]. Could PC production be a possible mechanism by which earthworms help detoxify arsenic? 

We tested this hypothesis by direct chemical analysis of phytochelatins in *L. rubellus* exposed to arsenic in the laboratory, and showed that phytochelatins were strongly arsenic-responsive. In addition, we also measured PCs in worms collected from mine sites with historical cadmium and arsenic contamination.

## Methods

### Ethics statement

Permission to sample for Alice Holt, Drayton, and Snowdon sites was given by, respectively, Forest Research, ADAS UK Ltd., and the Countryside Council for Wales. Permission was not required for the DGC and Shipham sites, as these are unmanaged and abandoned industrial land; *L. rubellus* is not a protected species.

### Arsenic exposure: laboratory samples

We took samples from a previously conducted study on the effects of soil arsenic on earthworm ecological parameters (population growth rate) [21], from which additional frozen powdered tissue was available for our tests here. The full experimental details will not therefore be repeated here, but briefly, *L. rubellus* was exposed for 28 days at a constant 13 °C to soil arsenic concentrations of 0, 3, 12, 36, and 125 mg kg^-1^ (n=5) in a clay loam soil. In addition, within the same experiment there were additional individual samples at concentrations of 2.4, 7.7, 9.6, 23, 29, 80, 100, and 156 mg kg^-1^ (n=1), i.e. there were 33 samples in total. 

### Field sampling from contaminated sites

We collected worms by digging and hand-sorting from four sites (n = 16 to 20 worms per site): Devon Great Consols, UK (DGC), an abandoned arsenic mine site; and a lead/zinc mine site near Shipham, UK, which also has elevated cadmium levels. Both sites had a nearby matched control site, where we attempted to match soil and site conditions as closely as possible. In addition, we collected a smaller number of worms (n = 3 or 4 per site) from three further control sites (Environmental Change Network sites), i.e. giving 7 sites in total: Alice Holt, Drayton, and Snowdon. The worms were briefly rinsed on-site, snap-frozen in liquid nitrogen, and returned to the laboratory on dry ice. We then extracted and analysed these for phytochelatins in the same way as for the laboratory-exposed samples (section 2.6). 

### Metal ion analysis

The soils from the field sites were air-dried and digested in hydrochloric-nitric acids using a microwave method involving heating 0.5 g of soil in 12 ml of aqua *regia* mix to 200°C according to United States Environmental Protection Agency protocol #3051A. Metal concentrations (Al, Ca, Ti, V, Cr, Mn, Fe, Co, Ni, Cu, Zn, As, Se, Mo, Cd, Sb, Ba, Hg, Pb) in these digests were determined on a Perkin Elmer Elan DRC II inductively coupled plasma mass spectrometry (ICP-MS) instrument using matrix-matched calibrants and Ga, In and Re as internal standards. Detection limits were defined as four times the standard deviation of five replicate blank determinations. A certified soil from the International Soil Exchange (ISE 921, Wageningen University, The Netherlands) was included in each batch as a quality control. Recoveries of certified values were within 15% in all cases. 

### Metabolite extraction and analysis by NMR spectroscopy

We extracted the frozen powdered tissue (80-100 mg) with 1 ml of ice-cold 60% acetonitrile, centrifuged the extracts (5 min, 16,000 *g*), and dried the supernatants under reduced pressure. We then analysed the samples by ^1^H nuclear magnetic resonance (NMR) spectroscopy: first, we reconstituted the extracts in ice-cold 0.65 ml of NMR buffer (100 mM phosphate buffer, pH 7.0, and 1 mM sodium trimethylsilyl-2,2,3,3-^2^H_4_-propionate (TSP), made up in ^2^H_2_O): the TSP served as an internal chemical shift reference, and the ^2^H_2_O both provided a field-frequency lock for the spectrometer and reduced the H_2_O signal. We then centrifuged the extracts again (16,000 *g*, 5 min, 4 °C), and transferred 0.6 ml to 5 mm NMR tubes, which were kept cold on ice until analysis, as earthworm tissue extracts are potentially affected by enzymatic changes even after extraction with organic solvents [22]. These were analysed on an Avance DRX600 spectrometer (Bruker, Rheinstetten, Germany) with a 14.1 T magnet and associated ^1^H resonance frequency of 600 MHz, using a 5 mm inverse geometry probe. The samples were kept at 300 K during analysis. We used the approach described by Beckonert et al. [23]; briefly, a presaturation sequence was used to remove the residual ^2^HOH signal, and the data acquired over 8 dummy scans and 128 scans with a recycle time of 5 s, giving a total acquisition time of around 11 min/sample. We processed the data in NMR Suite 6 (Chenomx, Edmonton, Canada), and used the same software to fit metabolites in a ‘targeted profiling’ approach [24]. The data were then normalized following the probabilistic quotient method of Dieterle et al. [25] on the fitted concentrations, i.e. the values were ultimately expressed as relative rather than absolute concentrations.

### Arsenic speciation analysis

This was based upon the approach of Button et al. [26], and analysed the proportion of arsenic found as As (III), As (V), arsenobetaine (AB), disodium methyl arsenate (MMA), and dimethylarsinic acid (DMA). We extracted dried homogenized earthworm tissue (around 1 g) by shaking in 10 ml of methanol:water (1:1 v/v) at 175 rpm for 4 h at 20 °C, centrifuged the extracts (1600 *g*, 10 min), and then dried the supernatants in a Syncore Analyst (Buchi, Zurich, Switzerland). We then reconstituted the dried extracts in 10 ml of deionised water and analysed them immediately. (We tested the stability of all of the analytes under these extraction conditions by separately spiking earthworm tissue: the recoveries of the spiked standards were on average 103.3%, with no evidence of interconversion between species.) For the speciation analysis, we used anion-exchange HPLC coupled to ICP-MS. The HPLC was a series 200 system and the mass spectrometer was an Elan DRC II (Perkin Elmer), connected with PEEK tubing and low dead volume connectors, and an automatic switching valve for easy column equilibration without overloading the ICP-MS system with eluent. The column was a Hamilton PRP-X100 (10 µm, 4.1 x 250 mm) with a reverse-phase guard column (RP-1, Phenomenex). We used a linear gradient for the chromatography, from 100% buffer A (4 mM NH_4_NO_3_, pH 8.7) to 100% buffer B after 10 minutes (60 mM NH_4_NO_3_, pH 8.7), at a flow rate of 1 ml min^-1^. This gave good separation of the five analytes. For the ICP-MS, we monitored ^75^As, with external calibrations for each As species in the range 0-250 μg l^-1^. The As (III) and As (V) standards were bought as aqueous 1000 mg l^-1^ stock solutions (Spex Certiprep), and AB, MMA, and DMA were from Sigma-Aldrich (Poole, UK). 

### Phytochelatin analysis

Standards (PC_2_ and PC_3_) were purchased from Cambridge Bioscience (Cambridge, UK). We extracted the tissue for the laboratory samples by bead-beating into three volumes of methanol containing 10 mM TCEP as a reducing agent, followed by centrifugation (5 min, 16,000 *g*). The field samples were extracted in a ratio of approx. 250 mg tissue (weighed accurately for each sample) to 2 ml of 1:2:2 v/v/v water:acetonitrile:methanol, as described in detail by Liebeke and Bundy [22], with TCEP added after extraction in a separate step. The supernatants were then derivatized with *N*-ethyl maleimide (freshly-prepared 266 mM solution was added to the extracts in a 1:50 v/v ratio) immediately before injection (from 2 to 30 μl) onto an HPLC system coupled to an API 2000 Q-TRAP mass spectrometer (AB Sciex, Warrington, UK). Chromatography was performed using an Ascentis Express (Sigma-Aldrich, Poole, UK) C18 column (100 x 3 mm), with gradient of 100% A (94.9% H_2_O: 5% acetonitrile: 0.1% formate) to 100% B (5% H_2_O: 94.9% acetonitrile: 0.1% formate) over 10 min. The flow rate was 200 μl/min. The MS was operated in negative mode using Turbo-Ionspray™ as the ion source and following the conditions: temperature 400°C, source gas 1 50 psi, ion source gas 2 60 psi, ion spray voltage -4500 V, curtain gas 40 psi, CAD gas setting 2; the DP (-25 V), EP (-9) and CEP (-2) were held constant for all transitions. Data were acquired and analysed using Analyst 1.4.2 software (Applied Biosystems). We monitored phytochelatins using four separate mass transitions: 790.2>201.2, 790.2>358.3, 709.2>661.4, and 790.2>304.3 for PC_2_ (retention time 5.7 minutes), and 1147.2>358.1, 1147.2>558.4, 1147.2>1018.4 and 1147.2>586.4 for PC_3_ (retention time 6.0 minutes). 

### Gene expression analysis

Total RNA was generated from arsenic exposed *Lumbricus rubellus* using an RNeasy Mini Kit and 500ng was used to generate complementary DNA by reverse transcription using a QuantiTect kit, both according to the manufacturer’s instructions (Qiagen, Manchester, UK). Expression profiles of the *pcs-1a* and *pcs-1b* genes, as well as the invariant internal control gene cyclophilin-B, were determined by quantitative PCR using an Mx3000™ instrument (Agilent, Cheadle, UK). The oligonucleotide primers for *pcs-1A* and *pcs-1B* (GenBank accession numbers KC981074 and KC981075 respectively) were 5’-TGCACTTTTCAGAGCTATGCAGCGA-3’ (*pcs-1A* forward), 5’-GCGAAAGATGAGACCGGGGCG-3’ (*pcs-1A* reverse), 5’-GAAAAGGATTTGGTGTTGATAATGGACGTGG-3’ (*pcs-1B* forward), and 5’-CAGTCTAAAGAGGACGGTCGGAAGC-3’ (*pcs-1B* reverse). Cyclophilin-B primers were taken from Sturzenbaum et al. [27]. The TaqMan real-time QPCR amplification reactions were performed in 25 μl reaction volumes in triplicate and consisted of 5 μl of template cDNA (1:10 dilution), and 12.5 μL of QuantiFast SYBR Green PCR mix (204054) containing 6-carboxyl-X-rhodamine (ROX) and oligonucleotide primers (1 μM). Amplification was carried out by thermal cycling at 95 °C for 5 min followed by 40 cycles of 95 °C for 15 seconds and 60 °C for 1 minute. The level of expression was measured using the C_T_ (threshold cycle) value, i.e., the cycle number at which the fluorescence (dRn) of each sample passes a threshold after correction for the baseline and ROX fluorescence. Values (average C_T_) of *pcs-1a* and *pcs-1b* expression in each cDNA sample were then normalized against the amount of cyclophilin-B in the same sample. The data were analyzed using the relative quantitation method (2^-ΔΔCT^) [28].

## Results and Discussion

### Phytochelatin2 increases in earthworms exposed to arsenic in the laboratory

**Figure 1:**
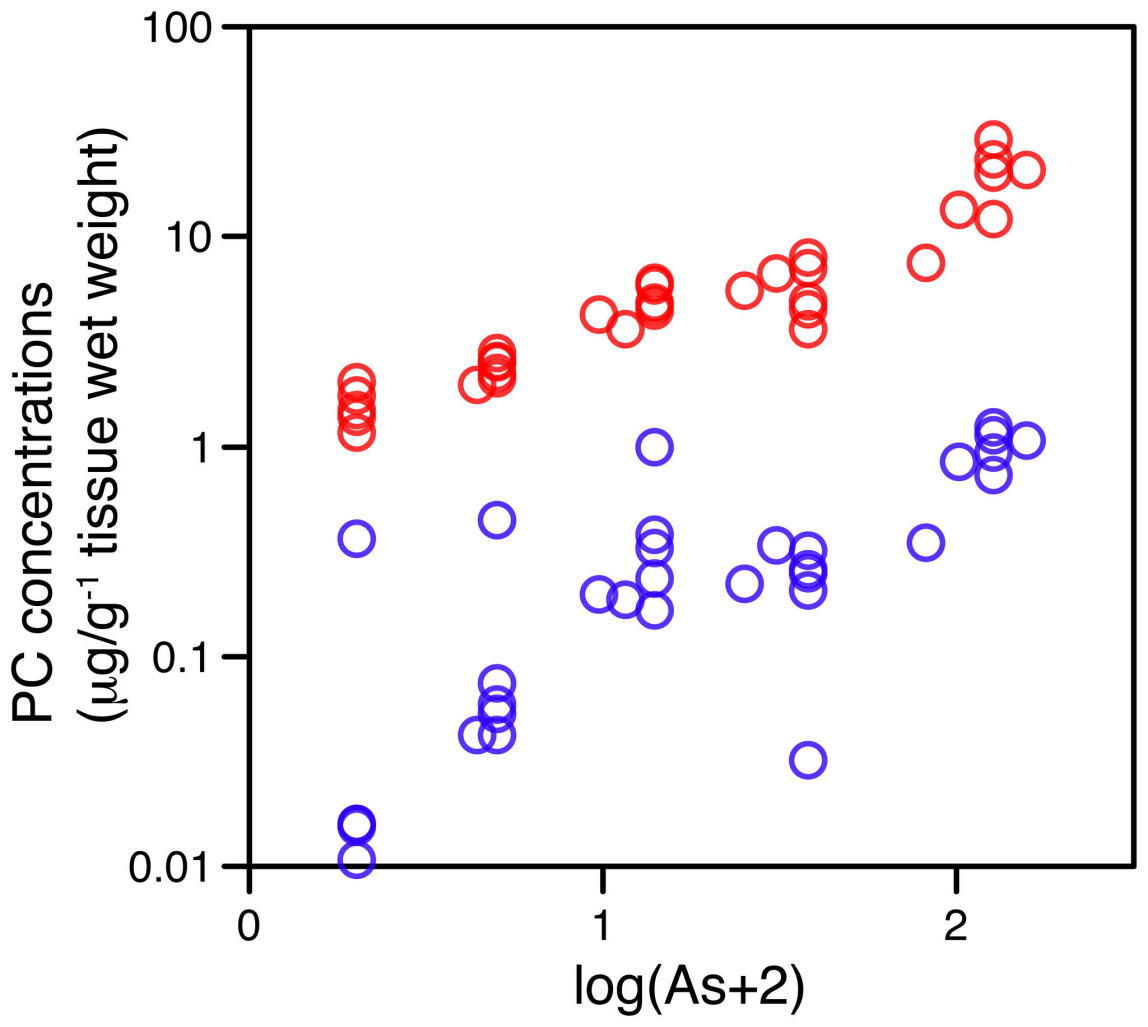
Phytochelatin-2 is present at higher levels than phytochelatin-3 in earthworms, and both are arsenic-responsive in laboratory exposures.

The samples of arsenic-exposed worms were taken from an earlier study by Anderson et al. [21], on the effects of arsenic on functional ecological parameters. They showed that the levels used did not kill the worms (although it did cause mortality at the highest concentration in an additional set of juvenile worms, not analysed here), but significantly reduced the cocoon production rate at the 125 mg kg^-1^ level, i.e. the amount of arsenic induced ecologically relevant sub-lethal effects at the highest replicated level tested. We used an LC-MS-MS method to monitor PCs, and also included a step to derivatize the free thiols to improve compound stability and chromatographic behaviour. PC_2_ was present at much higher levels than PC_3_; there was a very clear linear dose-response of PC_2_ to arsenic, with a rank correlation Spearmans ρ)of PC_2_ with soil arsenic of 0.95. PC_3_ also increased in response to arsenic, although with a more variable response ρ=, but was always lower than PC_2_ levels at corresponding arsenic concentrations (Figure 1).

### Changes in associated pathway metabolites

**Figure 2:**
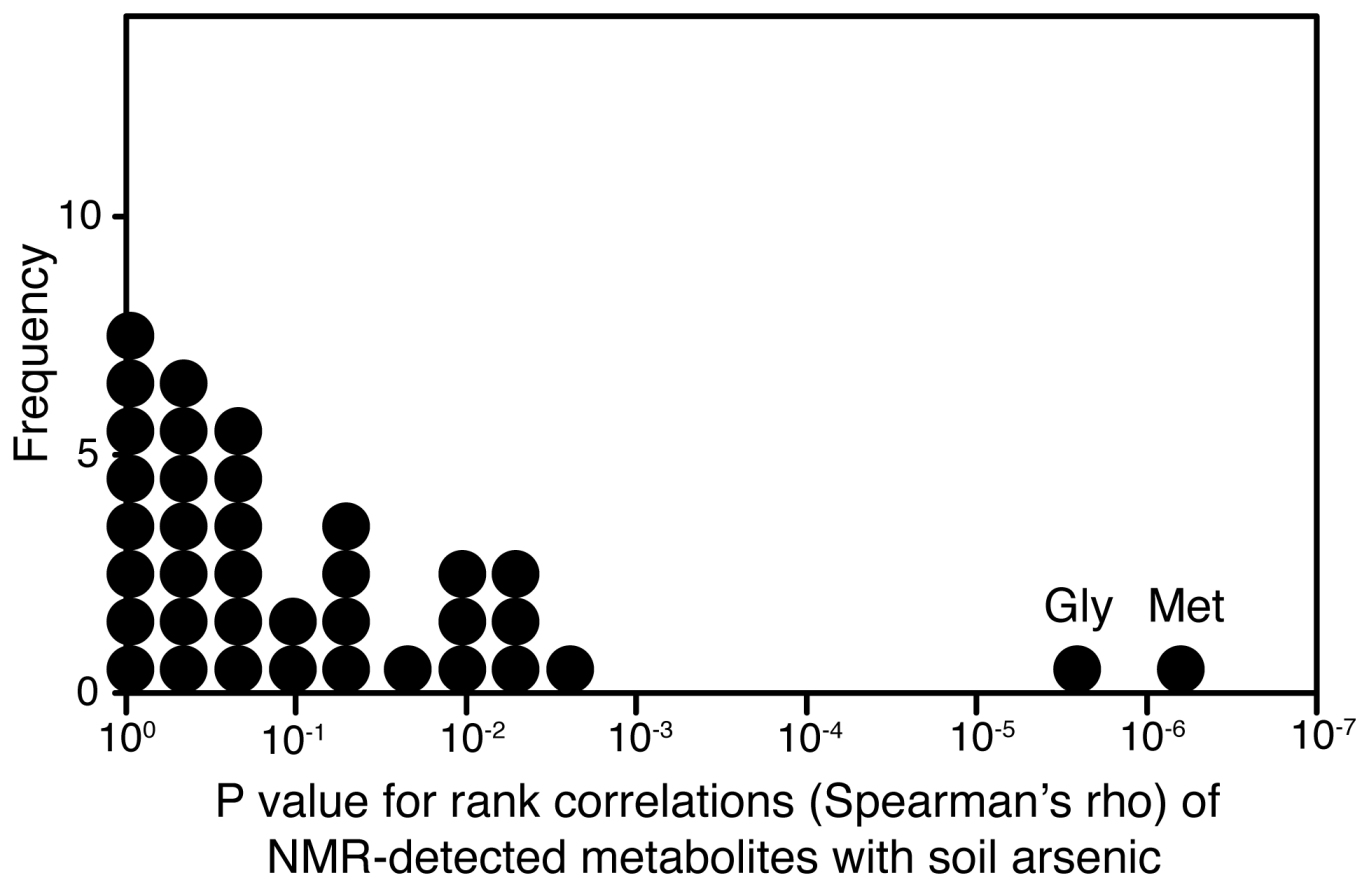
Glycine and methionine are the NMR-detectable metabolites most responsive to arsenic. Significance of rank correlations of all metabolites with soil arsenic concentrations (Spearman’s μ).

A previous metabolomic study of the responses of *C. elegans* to cadmium saw changes in cystathionine as well as phytochelatins [13]. Cystathionine is an intermediate in the transsulfuration pathway that interconverts homocysteine and cysteine, and thus generates cysteine from dietary methionine; the cysteine can then be fed into phytochelatin synthesis via glutathione. We wanted to see if there were similar metabolite alterations in *L. rubellus*, and so we carried out a separate untargetted NMR metabolomic analysis of the same samples used for PC analysis. In general, metabolomics can be thought of as providing a measure of biochemical phenotype, which can often be a valuable complement to existing biological data. The approach has already been widely used with ecologically relevant organisms, such as earthworms, for studying the phenotypic responses to a range of environmental factors, including toxic elements and anthropogenic pollution [29,30]. We have previously assigned NMR-visible metabolites in *L. rubellus* tissue extracts [31-33]. Here, we quantitated 36 metabolites from the NMR spectra (Table 1), and found that two metabolites were highly significantly associated with arsenic exposure (Spearman’s rank correlation): glycine and methionine (P < 10^-5^ and P < 10^-6^, respectively) (Table 1 and Figure 2). These are indeed both closely connected to the transsulfuration pathway: methionine is the starting point of sulfur assimilation and feeds directly into the pathway. Glycine is involved in one-carbon metabolism, and also has another potential interaction with the pathway to PCs as it is also used to form glutathione. These observations help confirm the biological pathways involved, and are a useful comparison to *C. elegans* responses to metal ions and PC induction. However, we could not tell whether cystathionine changed in earthworms as it did in *C. elegans* [13], as cystathionine was below the NMR detection limit in these samples.

**Table 1:** Metabolites identified and quantitated by NMR spectroscopy, and their association (Spearman’s rank correlation) with soil arsenic concentrations in laboratory-exposed worms.

	PubChem ID	Association with soil arsenic (ρ)
Glycine	750	0.75
Methionine	6137	0.73
Phenylalanine	6140	0.52
Succinate	1110	-0.50
Leucine	6106	0.48
Alanine	5950	0.47
HEFS**^*a*^**	n/a	0.46
Histidine	6274	-0.45
Valine	6287	0.44
Tyrosine	6057	0.40
Lysine	5962	0.37
Isoleucine	6306	0.36
scyllo-Inositol	892	-0.34
Glutamate	33032	0.34
Lactate	612	-0.27
N,N-Dimethylhistidine	440274	-0.27
Threonine	6288	0.25
Tryptophan	6305	0.25
Pyruvate	1060	0.23
myo-Inositol	892	-0.22
Phosphocholine	1014	0.20
Choline	305	0.18
Fumarate	444972	0.17
Malate	525	0.17
Asparagine	6267	0.16
Maltose	6255	-0.15
AMP	6083	-0.14
Aspartate	5960	0.14
UDP-N-acetylglucosamine	445675	-0.08
Lombricine	439556	0.07
Betaine	247	0.06
Glucose	5793	0.04
Glutamine	5961	-0.02
Acetate	176	0.02
Nicotinate	938	0.02
ADP	6022	-0.01

a: 2-hexyl-5-ethyl-furan-3-sulfonate [31].

### No biological transformation of arsenic

Previous studies of arsenic speciation in earthworms have largely focussed on wild populations naturally occurring at mine sites, and have shown that extractable arsenic species are dominated by inorganic arsenic [26,34]. We wanted to compare if this were also true for laboratory exposures of naïve worms, and used liquid chromatography with arsenic-specific detection to measure speciation into five main components – arsenic (III), arsenic (V), AB, MMA, and DMA – and analysed samples from the control worms plus worms from the two highest replicated concentrations (36 mg kg^-1^ and 125 mg kg^-1^). The total extractable (soluble) arsenic was not significantly different between the control and 36 mg kg^-1^ worms, but the 125 mg kg^-1^ worms had significantly more soluble arsenic than either of the other two groups (P < 0.01; one-way ANOVA, Fisher’s LSD test). We only detected DMA in one sample, and that at a very low level (

< 0.5% of extractable arsenic), so we decided not to present the results for DMA. The large majority of the arsenic was present as inorganic arsenic, and the proportion of inorganic arsenic increased with increasing soil concentration, from 92% for the control worms (which contained low concentrations of arsenic from residual exposure to background levels in the control soil) to >99% for the highest concentration (Table 2). In addition, the proportion present as arsenic (III) increased with soil concentration: the inorganic arsenic was entirely present as arsenic (V) for the control worms, while at 125 mg kg^-1^, more than one-fifth was present as arsenic (III). The organoarsenic compounds AB and MMA were present only as small proportions within the exposed earthworms (Table 2). We did not detect any arsenosugars in our samples, which mirrors the findings of a laboratory study by Button et al. [35], supporting their suggestion that these compounds may be accumulated by earthworms from soil rather than endogenous products. Clearly, there is no evidence for arsenic detoxification by forming organoarsenic species.

**Table 2:** Biological arsenic speciation at different soil arsenic concentrations; data presented as % of total soluble arsenic ± SD (n = 5). AB: arsenobetaine. MMA: monomethylarsenate.

Soil arsenic added (mg kg^-1^)	Tissue arsenic (total) (mg kg^-1^) **^*a*^**	Tissue arsenic (soluble) (relative units)	As(V)	As(III)	AB	MMA
0	0.7 ± 0.3	33 ± 17	91.7% ± 5.8	nd	0.4% ± 0.4	7.9% ± 5.9
36	5.8 ± 0.8	22 ± 4	89.9% ± 2.7	6.0% ± 5.5	3.6% ± 3.8	0.5% ± 0.7
125	26.2 ± 4.0	63 ± 14	78.3% ± 4.9	21.6% ± 4.9	0.2% ± 0.2	nd

a: data originally published in Anderson et al. [21], ± SD (n = 5).

### Phytochelatin synthase expression was not altered by arsenic treatment in *Lumbricus rubellus*

PCS regulation in eukaryotes is often complex with multiple regulatory steps, including post-transcriptional regulation [36]. The limited evidence to date tends to suggest that PC biosynthesis is not primarily transcriptionally regulated in metazoans. Challenging the model organism *C. elegans* with cadmium across a range of concentrations does not lead to clear increases in *pcs-1* gene expression [37], even though phytochelatin synthesis is critical in protecting against cadmium toxicity [11,13]. Vandenbulcke and colleagues have monitored the earthworm *Eisenia fetida* PCS expression in response to contaminated field soils, and generally found little or no association with metals [38,39]. 

**Figure 3:**
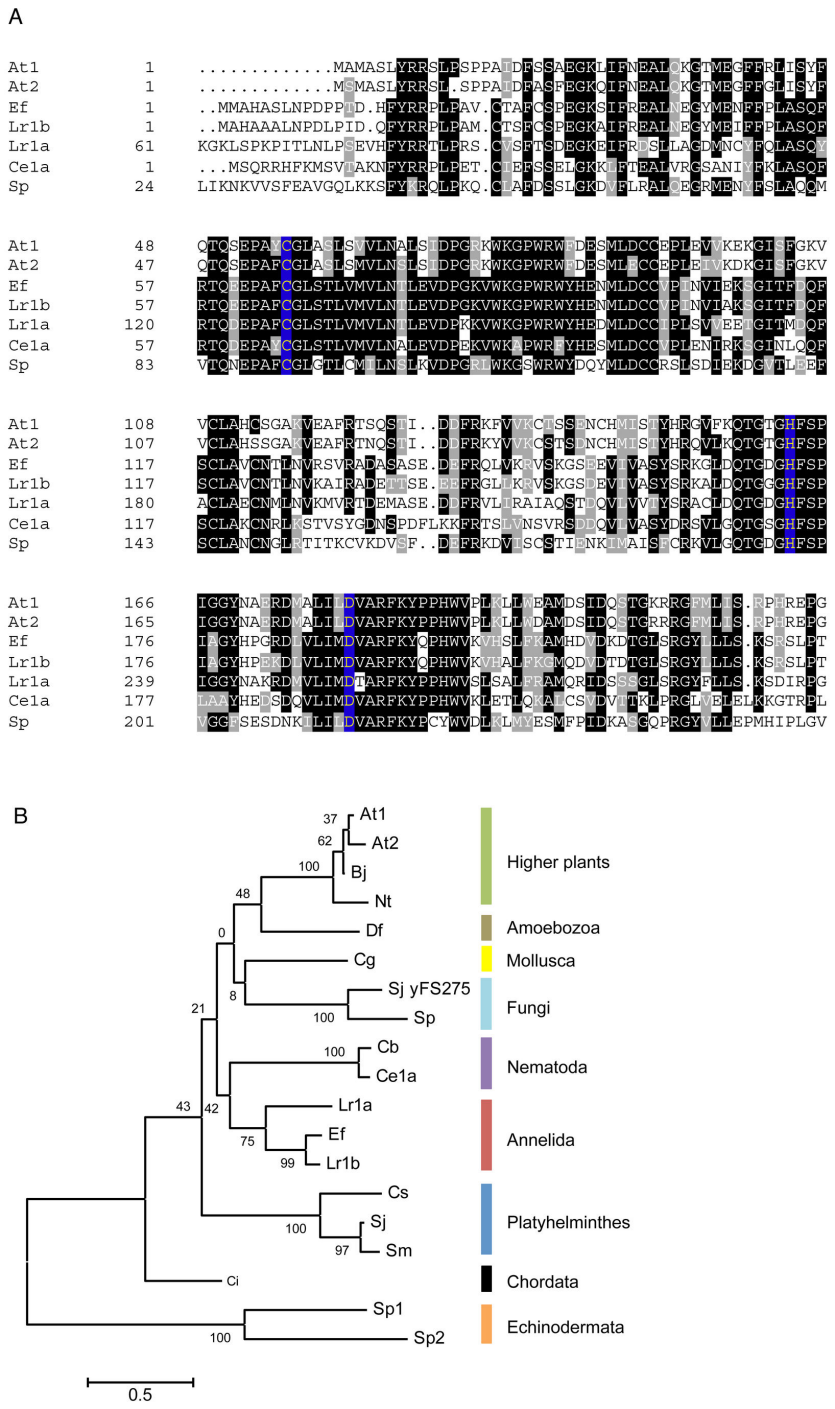
Earthworm phytochelatin synthase sequences compared to phytochelatin synthase sequences from other species. A: alignment of *Lumbricus rubellus* phytochelatin synthase sequences (conserved N-terminal region only, full sequence shown in Figure S1) against key species with validated PCS activity (*Arabidopsis thaliana* PCS1 and PCS2, *Eisenia fetida*, *L. rubellus* PCS1a and PCS1b, *Caenorhabditis elegans*, and *Schizosaccharomyces pombe*). Conserved residues are shown with white text on black background; the conserved Cys-His-Asp catalytic triad is shown with yellow on blue background. B: Phylogenetic tree of phytochelatin synthase genes. The evolutionary history was inferred by using the Maximum Likelihood method based on the Whelan and Goldman model [48]. The tree with the highest log likelihood (-3323.4018) is shown. The percentage of trees in which the associated taxa clustered together is shown next to the branches. Initial tree(s) for the heuristic search were obtained by applying the Neighbor-Joining method to a matrix of pairwise distances estimated using a JTT model. A discrete Gamma distribution was used to model evolutionary rate differences among sites (5 categories (+G, parameter = 1.9794)). The tree is drawn to scale, with branch lengths measured in the number of substitutions per site. The analysis involved 19 amino acid sequences. All positions containing gaps and missing data were eliminated. There were a total of 126 positions in the final dataset. Evolutionary analyses were conducted in MEGA5 [49]. At – *A. thaliana*. Bj – *Brassica juncea*. Nt – *Nicotiana tabacum*. Df – *Dictyostelium fasciculatum*. Cg – *Crassostrea gigas*. Sj yFS275 – *Schizosaccharomyces japonicum*. Sp – *S. pombe*. Cb – *C. briggsae*. Ce1a – *C. elegans*. Lr1a and Lr1b – *Lumbricus rubellus* PCs 1a and 1b respectively. Cs – *Clonorchis sinensis*. Sj – *Schistosoma japonicum*. Sm – *Schistosoma mansoni*. Ci – *Ciona intestinalis*. Sp1 and Sp2 – *Strongylocentrotus purpuratus*.

Hence, we predicted that *L. rubellus* PCS expression would, similarly, have a weak or no response to arsenic. In order to test this, we measured PCS expression levels using qPCR, but this first required an initial characterization of the gene sequence, which has not been described before for *L. rubellus*. We carried this out using existing genome sequence data, and discovered that *L. rubellus* has two PCS genes, *pcs-1A* and *pcs-1B*, the first metazoan so far known to do so. We performed an alignment and clustering of a number of PCS gene sequences; the *L. rubellus* PCS sequences were clearly highly conserved when compared to other key PCS proteins (plant, fungal, and animal) with validated phytochelatin biosynthetic enzyme activity (Figure 3A and Figure S1, supplementary online information). This includes the Cys-His-Asp catalytic triad [40]. When compared to a wider range of PCS proteins, including some from metazoans which have not yet had PCS activity validated, the earthworm PCS (*L. rubellus* and *E. fetida*) formed a separate cluster, most closely related to nematode PCS (Figure 3B). Intriguingly, the *L. rubellus pcs-1B* clustered more closely with the *E. fetida* PCS than with the *pcs-1A*, indicating an ancient divergence of the two *L. rubellus* paralogs. 

**Figure 4:**
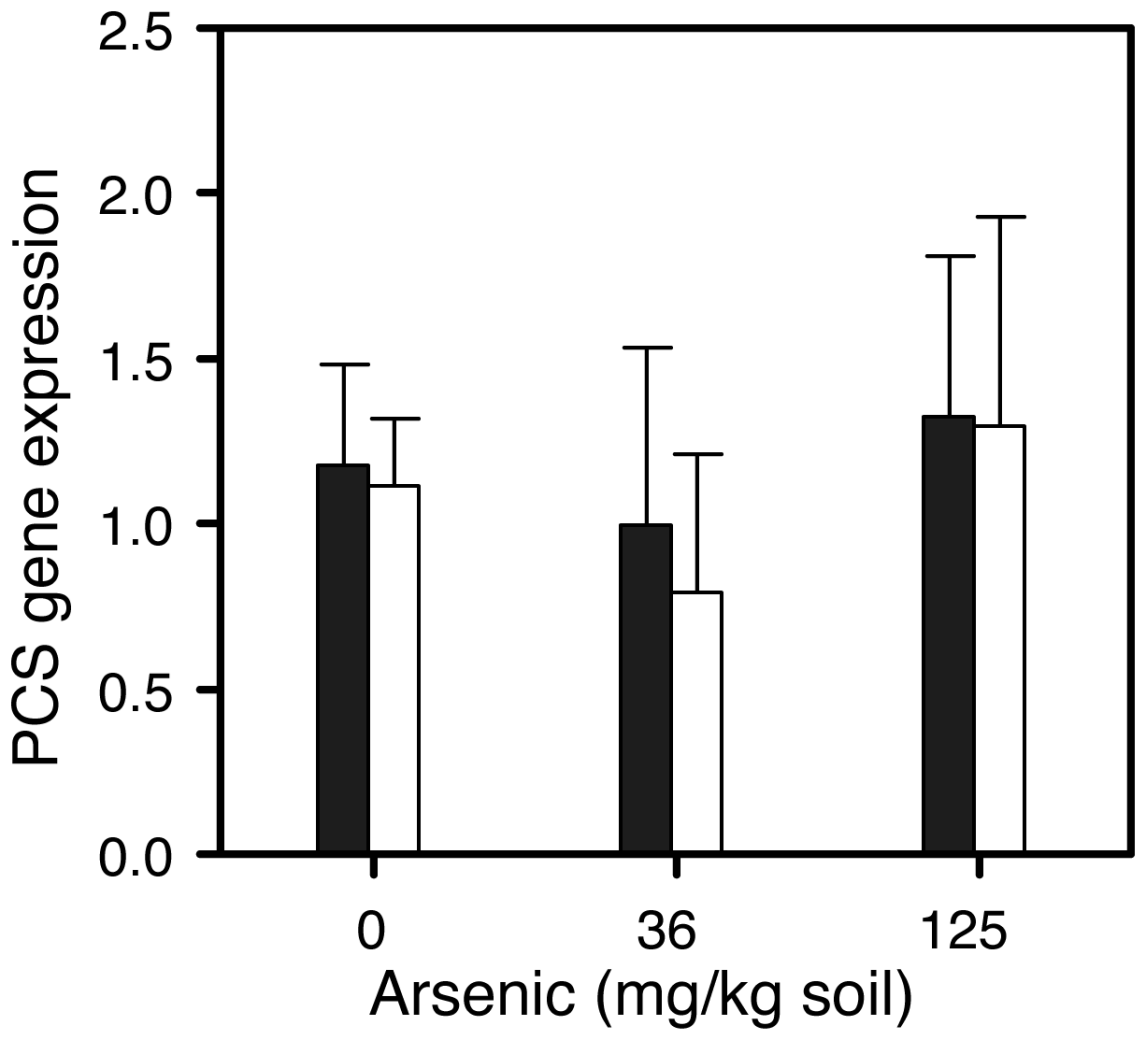
Lumbricus rubellus phytochelatin synthase isoforms were not arsenic responsive at the concentrations and exposure duration tested. Filled bars: *pcs-1a*. Empty bars: *pcs-1b*. Error bars represent 95% confidence intervals (n = 5).

We then used the gene sequence information to design primers for qPCR analysis of gene expression. There were no significant differences between the control data and either the medium (36 mg kg^-1^) or high (125 mg kg^-1^) arsenic level worms, for either *pcs-1A* or *pcs-1B* (Figure 4). It appears that, for earthworms, monitoring PCS gene expression may not be sufficient to tell whether functional responses to metal pollution have occurred, and it will be necessary to measure PCs directly. However, studies on closely-related soil oligochaetes have shown that gene expression in response to toxic challenges may peak after shorter times than the 28 day period used in our current study [41]. Furthermore, *Eisenia fetida* PCS expression was cadmium responsive at low soil concentrations only (8 mg kg^-1^ soil), with no difference from controls at higher cadmium levels (80 and 800 mg kg^-1^) [19]. It would be interesting in the future to carry out a fuller characterization of earthworm phytochelatin responses, i.e. testing a wide concentration range for both cadmium and arsenic, and monitoring PCS gene expression, protein levels, and PC levels across time.

### Phytochelatin analysis in field samples

**Figure 5:**
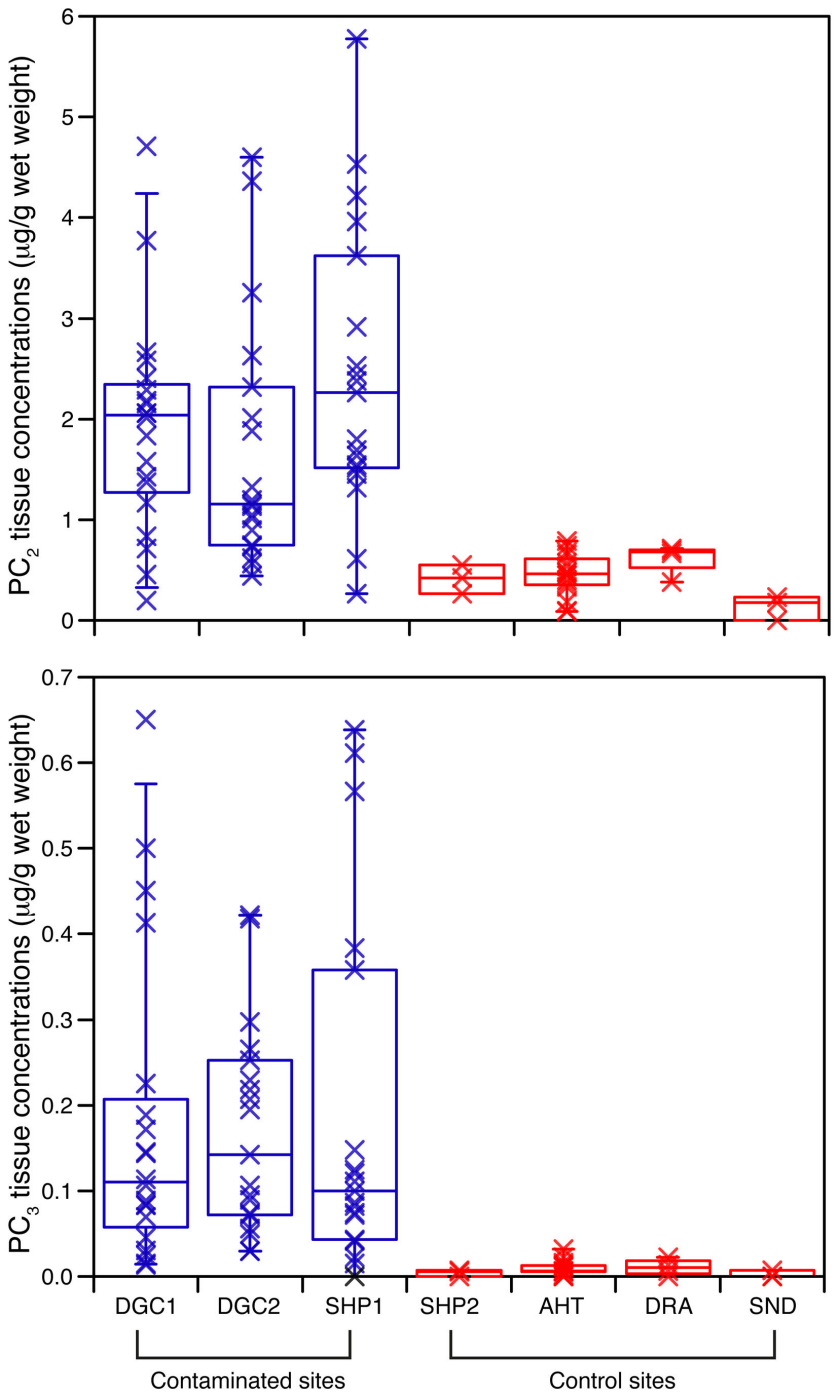
Phytochelatins are elevated in worms from contaminated field sites (blue) compared to worms from relatively pristine control sites (red). A: phytochelatin-2; B: phytochelatin-3. The boxes display the median, and first and third quartile boundaries, and the whiskers represent 95% confidence intervals. DGC1: Devon Great Consols ‘control’ site, in effect a second arsenic-contaminated site. DGC2: Devon Great Consols contaminated site. SHP1: Shipham contaminated site. SHP2: Shipham control site. AHT: Alice Holt. DRA: Drayton. SND: Snowdon.

As discussed above, previous studies found little or no response of earthworm PCS gene expression to contaminated field soils. Given the clear response of PC production (but not gene expression) to arsenic in our current study, we wanted to know if PC levels were increased in natural populations of worms exposed to potentially toxic ions. We sampled *L. rubellus* from an arsenic contaminated site (DGC) and from a predominantly cadmium-contaminated site (Shipham), plus matched control sites, and measured levels of both PC_2_ and PC_3_ (Figure 5). Soil metal concentrations for all sites are given in Table 3.

**Table 3:** Field site data including metal ion concentrations (mg kg^-1^ soil).

	DGC1	DGC2	Shipham 1	Shipham 2	Alice Holt	Drayton	Snowdon
Status	Contaminated	Contaminated	Contaminated	Control	Control	Control	Control
OS Grid Reference	SX 42626 73976	SX 42633 73289	ST 44815 57429	ST 46921 59370	SU 79793 40144	SP 16299 55056	SH 63864 55111
Latitude	50.544322	50.538145	51.313283	51.330935	51.154994	52.19350433	53.075826
Longitude	-4.2224836	-4.2220974	-2.7931634	-2.7632397	-0.86044997	-1.76297617	-4.0336132
Soil pH	5.49	5.3	6.33	5.38	4.93	6.54	6.04
Soil organic carbon	17.2	29.7	33	11.7	13.2	17.1	13.9
Land use	pasture	minesite	minesite	pasture	Woodland	pasture	pasture
P	1150	286	2010	992	365	2010	531
S	756	1600	1810	575	404	1190	687
Ca	1690	3390	10500	3440	1650	35000	1020
Ti	38	41	61.2	53.6	7.21	34.1	1520
V	39.5	38.5	29.9	34.9	24.3	31.8	238
Cr	32	17.8	21.9	22.9	12.8	15.5	78.1
Mn	568	609	2620	455	55.8	817	1350
Fe	47400	79600	75000	26500	16300	28200	69900
Co	16	23.4	12.9	7.48	5.96	4.86	30
Ni	24.2	23.6	31.6	16.4	10.1	10.9	30
Cu	101	2610	60	18.3	12.7	41.6	17.5
Zn	127	264	19300	320	42.5	182	114
As	321	6250	681	37.5	13	25.8	17.6
Se	1.98	2.56	2.39	0.457	0.491	0.501	1.4
Mo	1.1	1.2	5.28	0.704	0.278	1.01	0.522
Cd	0.172	0.215	169	2.05	0.1	0.416	0.305
Sb	1.41	13.1	32.3	0.894	0.42	0.626	0.511
Ba	45.3	44.5	662	304	19.6	47.8	8.51
Hg	0.067	0.746	9.74	0.066	0.156	0.069	0.145
Pb	70.4	202	4940	162	26.5	30.3	36.1

This definitively confirmed that PC levels are elevated in natural populations, and hence that PC synthesis is likely an important real-world earthworm detoxification mechanism. There was a very clear upregulation of PCs at the Shipham site compared to the control; PC_2_ levels were, as for the laboratory study, considerably higher than PC_3_, but the relative increase was greater for PC_3_. The PC levels were high at both the DGC control and contaminated sites; however, the soil arsenic concentration of the DGC ‘control’ site (321 mg kg^-1^) was well above the levels that caused PC induction in the lab samples, and examining the tissue arsenic levels showed that the DGC ‘control’ worms had as much arsenic as the Shipham contaminated site worms (supporting information, Figure S2). In other words, our ‘control’ DGC site was not actually a good choice of control at all, and so we obtained worms from three other well-characterized Environmental Change Network sites [42], that we were confident were good controls (Alice Holt, Drayton, and Snowdon); there were fewer worms per site for this follow-up experiment, but still sufficient to confirm that the PC_2_ and PC_3_ levels were consistently low for these control sites – very similar to the Shipham control site – and that the DGC sites and the Shipham contaminated site had clearly elevated phytochelatin levels. It should also be noted that the Shipham contaminated site had very high levels of both cadmium and zinc, but the arsenic levels were also elevated (about twice as much arsenic as for the DGC ‘control’ site, although only about one-tenth as much for the DGC contaminated site), and so we cannot conclude from this one site whether the *L. rubellus* PCs were increased at Shipham in response to cadmium, zinc, arsenic, or a combination of all three. 

The field samples in general had lower phytochelatin levels (for both PC_2_ and PC_3_) than the laboratory worms: the highest tissue concentration of PC_2_ was 29.3 μg g^-1^ tissue wet weight for the laboratory-exposed worms (with an average value of 21 μg g^-1^ for the highest replicated exposure level), and 5.8 μg g^-1^ for the field worms. We do not know why this should be the case, but we suspect that it may be because of differences between a relatively short-term laboratory experiment, and chronic long-term exposure for natural populations. It is also unlikely that our laboratory worm population is genetically identical to the field populations.

### Wider significance

What implications do these findings have? Our results do not directly demonstrate a direct role of PCs in arsenic detoxification in earthworms, and the fact that earthworms are not model organisms means that it is much harder to prove this than for other, more genetically tractable species such as *C. elegans*. However, we think it is likely. The final total amounts of PC_2_ are of the order of several μg in arsenic-exposed earthworms, assuming a typical body weight of around 1 g for *L. rubellus*. Phytochelatins only bind to As(III), not As(V) [43]. In the laboratory worms, there was 5.6 mg kg^-1^ As(III) and 20.5 mg kg^-1^ As(V) on a dry weight basis; assuming that 85% of the tissue is water [44], then this would result in approximate tissue concentrations of 11 nmol g^-1^ and 41 nmol g^-1^ for As(III) and As(V) respectively. Given that the PC_2_ tissue concentrations were 39 nmol g^-1^ for the highest replicated arsenic exposure level, there is clearly scope for it to play a role in helping detoxify at least As(III) – the more toxic arsenic species. The situation is unclear for the field worms, which have lower PC concentrations than the lab worms (an average of about 4 nmol g^-1^ PC_2_ for worms from contaminated sites); we also lack arsenic speciation data for these samples.

There is a large body of work on metal handling mechanisms in invertebrates, with applications to understanding how they are affected by environmental pollution, but also for monitoring biomarker responses to pollution. Probably the most-studied set of molecules is metallothioneins, a group of small, cysteine-rich metal-binding proteins, that are found in all living things [45]. These are often highly metal-responsive in laboratory tests in many animal species [46], including earthworms [47]. As a result, studies on animals in metal-contaminated environments often focus on metallothionein responses. In contrast, there has been almost no study of the possible role of PCs in the responses of ecologically relevant animal species to metal contamination. PCs are more important than metallothioneins in protecting against cadmium for the nematode *C. elegans* [12,13]. Could the same be true for other species, and hence of general importance for ecotoxicologists [46]?

Species with PCS genes are spread across almost the whole metazoan tree of life. On the other hand, some important taxa have apparently lost PCS – e.g. the phylum Arthropoda, and the sub-phylum Craniata [17]. In addition, not all enzymes with PCS homology will necessarily produce PCs: for example, the S. *mansoni* PCS is functional when cloned into an appropriate host, but may not produce PCs *in vivo* in response to metals [15]. Nonetheless, the distribution of the PCS gene across many phyla argues that it is likely to be found in more animal species in the future. 

##  Conclusion

Phytochelatins have now been shown to be responsive to toxic elements in two separate animal phyla, and may well prove to be of importance in ecologically-relevant invertebrates for detoxifying soft metal ions. The evidence to date suggests that monitoring PC levels directly is more reliable than monitoring gene expression; PCs could potentially be used as biomarkers for earthworm responses to metal contamination, although further knowledge of their baseline variability in natural populations is needed for this to be achieved. 

## Supporting Information

Figure S1 **Alignment of PCS sequences for *Arabidopsis thaliana* (At1 and At2), *Eisenia fetida* (Ef), *Lumbricus rubellus* (Lr1b and Lr1a), *Caenorhabditis elegans* (Ce1a), and *Schizosaccharomyces pombe* (Sp).**
(DOCX)

Figure S2 **The Devon Great Consols** ʻcontrolʼ site (DGC1) has elevated tissue arsenic levels over the other control sites, and should be considered as a contaminated not control site.
(TIFF)
