# Supplementary material for: Earthworms Produce phytochelatins in Response to Arsenic
Source: PLoS One. 2013 Nov 22;8(11):e81271. doi: 10.1371/journal.pone.0081271 (PMC3838358; doi:10.1371/journal.pone.0081271)
Supplement: Figure S1 — Alignment of PCS sequences for Arabidopsis thaliana (At1 and At2), Eisenia fetida (Ef), Lumbricus rubellus (Lr1b and Lr1a), Caenorhabditis elegans (Ce1a), and Schizosaccharomyces pombe (Sp). (DOCX) [file pone.0081271.s001.docx]

At1 1 ............................................................
At2 1 ............................................................
Ef 1 ............................................................
Lr1b 1 ............................................................
Lr1a 1 MSKATLHTLRIARQLRSSKDCHPWCQIPQLVTVALVLNFSIGVRVTSTGREPNTTQPFAN
Ce1a 1 ............................................................
Sp 1 ....................................MNI.VKRAVPELLRGMTNATPNIG

At1 1 .............MAMASLYRRSLPSPPAIDFSSAEGKLIFNEALQKGTMEGFFRLISYF
At2 1 .............MSMASLYRRSL.SPPAIDFASFEGKQIFNEALQKGTMEGFFGLISYF
Ef 1 ..MMAHASLNPDPPTD.HFYRRPLPAV.CTAFCSPEGKSIFREALNEGYMENFFPLASQF
Lr1b 1 ..MAHAAALNPDLPID.QFYRRPLPAM.CTSFCSPEGKAIFREALNEGYMEIFFPLASQF
Lr1a 61 KGKLSPKPITLNLPSEVHFYRRTLPRS.CVSFTSDEGKEIFRDSLLAGDMNCYFQLASQY
Ce1a 1 ...MSQRRHFKMSVTAKNFYRRPLPET.CIEFSSELGKKLFTEALVRGSANIYFKLASQF
Sp 24 LIKNKVVSFEAVGQLKKSFYKRQLPKQ.CLAFDSSLGKDVFLRALQEGRMENYFSLAQQM

At1 48 QTQSEPAYCGLASLSVVLNALSIDPGRKWKGPWRWFDESMLDCCEPLEVVKEKGISFGKV
At2 47 QTQSEPAFCGLASLSMVLNSLSIDPGRKWKGPWRWFDESMLECCEPLEIVKDKGISFGKV
Ef 57 RTQEEPAFCGLSTLVMVLNTLEVDPGKVWKGPWRWYHENMLDCCVPINVIEKSGITFDQF
Lr1b 57 RTQEEPAFCGLSTLVMVLNTLEVDPGKVWKGPWRWYHENMLDCCVPINVIAKSGITFDQF
Lr1a 120 RTQDEPAFCGLSTLVMVLNTLEVDPKKVWKGPWRWYHEDMLDCCIPLSVVEETGITMDQF
Ce1a 57 RTQDEPAYCGLSTLVMVLNALEVDPEKVWKAPWRFYHESMLDCCVPLENIRKSGINLQQF
Sp 83 VTQNEPAFCGLGTLCMILNSLKVDPGRLWKGSWRWYDQYMLDCCRSLSDIEKDGVTLEEF

At1 108 VCLAHCSGAKVEAFRTSQSTI..DDFRKFVVKCTSSENCHMISTYHRGVFKQTGTGHFSP
At2 107 VCLAHSSGAKVEAFRTNQSTI..DDFRKYVVKCSTSDNCHMISTYHRQVLKQTGTGHFSP
Ef 117 SCLAVCNTLNVRSVRADASASE.DEFRQLVKRVSKGSEEVIVASYSRKGLDQTGDGHFSP
Lr1b 117 SCLAVCNTLNVKAIRADETTSE.EEFRGLLKRVSKGSDEVIVASYSRKALDQTGGGHFSP
Lr1a 180 ACLAECNMLNVKMVRTDEMASE.DDFRVLIRAIAQSTDQVLVVTYSRACLDQTGDGHFSP
Ce1a 117 SCLAKCNRLKSTVSYGDNSPDFLKKFRTSLVNSVRSDDQVLVASYDRSVLGQTGSGHFSP
Sp 143 SCLANCNGLRTITKCVKDVSF..DEFRKDVISCSTIENKIMAISFCRKVLGQTGDGHFSP

At1 166 IGGYNAERDMALILDVARFKYPPHWVPLKLLWEAMDSIDQSTGKRRGFMLIS.RPHREPG
At2 165 IGGYNAERDMALILDVARFKYPPHWVPLKLLWDAMDSIDQSTGRRRGFMLIS.RPHREPG
Ef 176 IAGYHPGRDLVLIMDVARFKYQPHWVKVHSLFKAMHDVDKDTGLSRGYLLLS.KSRSLPT
Lr1b 176 IAGYHPEKDLVLIMDVARFKYQPHWVKVHALFKGMQDVDTDTGLSRGYLLLS.KSRSLPT
Lr1a 239 IGGYNAKRDMVLIMDTARFKYPPHWVSLSALFRAMQRIDSSSGLSRGYFLLS.KSDIRPG
Ce1a 177 LAAYHEDSDQVLIMDVARFKYPPHWVKLETLQKALCSVDVTTKLPRGLVELELKKGTRPL
Sp 201 VGGFSESDNKILILDVARFKYPCYWVDLKLMYESMFPIDKASGQPRGYVLLEPMHIPLGV

At1 225 LLYTLSCKDESWIE...IAKYLKEDVPRLVSSQHVDSVEKIISVVFKSLPSNFNQFIRWV
At2 224 LLYTLSCKDESWIS...IAKYLKEDVPRLVSSQHVDTIERILYVVFKSLPANFNQFIKWM
Ef 235 VLFRLTS......N...LCVNGVQ..STNVVRFITDWKEWLVTS.....ASSTNSNKILD
Lr1b 235 VLFRLTA......N...LCVNGVQ..STKVVQFITDWKSWLSTP.....TNSPNSNKALD
Lr1a 298 LIFRLSH......H...LSPMYLQNNSSQISTFVQSWERQIMTRVDVDCSTSDRSGVVVR
Ce1a 237 IMYGLKA..............YVNINDSDFATSVISWNQFLLCDPLEDDEEEFQ..LCCR
Sp 261 LTVGLNKYSWRNVSKHILQQAATVKNADNLAEILLSINQSSIPLIQERSNSSKSGDFEHF

At1 282 AEIRITEDSNQNLSAEEKSRLKLKQLVLKEVHETELFKHINKFLSTVGYEDSLTYAAAKA
At2 281 AEIRRTEDVNQNLSSEEKSRLKLKQELLKQVQETKLFKHVDKFLSSV.YEDNLPYVAAKV
Ef 279 TAVNQLVKFLN.LKES.NLSMALVEVVGFENISMEHKYAIKQILEAL..KNLPVYQVVRS
Lr1b 279 TAVNQLVKFLN.LNEELSLMMALVEVVGFESISAEHRSAIRQILEAL..KNLPVYQVVRK
Lr1a 349 LAVSRLLSLLNELDSSTFLLTAQADPTFSSEHSPLYGRDVRKMLSSM..ESLPIFHAIQE
Ce1a 281 KFGQCFAPHAMCCTQKTFDADQKNSCTECSTDQNEACKMICSEIRRTRFAEVFSSSAVAA
Sp 321 KECIRSTKTYHLFLKHTNTNVEYITMAFWAIFSLPMIQKALPKGVLEEIQSLLKEVEISE
At1 342 CCQGAEILSGSPSKEFCCRETCVKCIKGPDDSEGTVVTGVVVRDGNEQKVDLLVPSTQTE
At2 340 YCDGDEILSGYESDESCCKETCVKCIKGLGEEKVTVVA......................
Ef 335 CIPGGDKLESETH...VPVDDGPHPEKFSMKPKTW.........................
Lr1b 336 YLPGGDKFESEMH...IPVHDDPHGENFSLKLKSW.........................
Lr1a 407 GVLSGKFPQASLEKAGVPGQSSSSLAGFEHTDVAYSVSSANLPCLRIN............
Ce1a 341 LLIAWPFEKGYSERSDRIGNLAEKYKNEFSAETMNEMN......................
Sp 381 INTQLTALKKQLDSLTHCCKTDTGCCSSSCCKNT..........................

At1 402 CECGPEATYPAGNDVFTALLLALPPQTWSGIKDQALMHEMKQLISMASLPTLLQEEVLHL
At2 378 ........YPSGNDVFTALLLALPPQTWSGIKDQSLLQEMKQLISMVSHPTLLQQEVLHL
Ef 367 .............HYVTMLLLMWPYRDDEGS.FGRCLDQLMADQLKASASELLQNEVTIL
Lr1b 368 .............HYVAMVLLIWPYEDTKGS.FGASLDRLMADQVKGSASELLQNEVTIL
Lr1a 455 ...ANVVESVTAVHFLTMLILAWPYHHCGGERLCTELHRYTSVLLSRPDLELLKNEVARL
Ce1a 379 .........QLTTQIRTLISCSKPPVVININKPDATSNKCCKNKIGQSCACANDVNL...
Sp ............................................................

At1 462 RRQL.QLLKRCQENKEEDDLAAPAY.....
At2 430 RRQL.EMLKRCQENKEDEELSAPA......
Ef 413 RQQVSSVLGFV.AVCDACPCKQKAAEAAK.
Lr1b 414 RQQVSSVLGYV.AVCTTCTCKQKAPEEPAK
Lr1a 512 RQQLTSILSYCKSCCHEDGCK.........
Ce1a ..............................
Sp ..............................
