# Supplementary figures and images for: Earthworms Produce phytochelatins in Response to Arsenic
Source: PLoS One. 2013 Nov 22;8(11):e81271. doi: 10.1371/journal.pone.0081271 (PMC3838358; doi:10.1371/journal.pone.0081271)

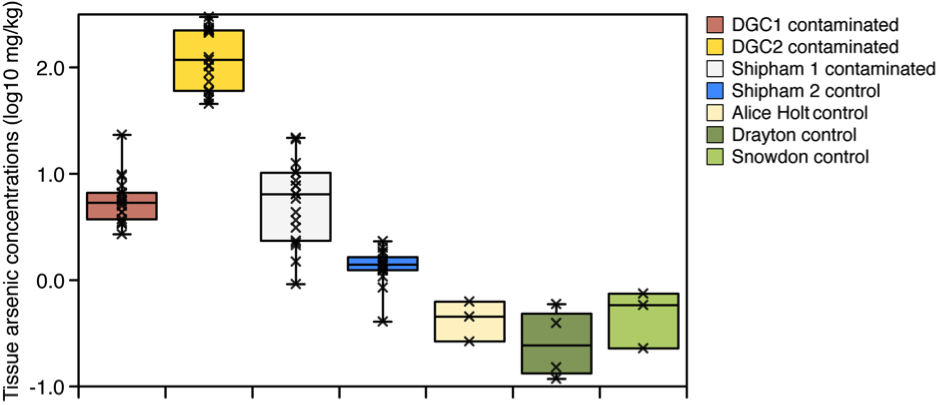

Supplement: Figure S2 — The Devon Great Consols ʻcontrolʼ site (DGC1) has elevated tissue arsenic levels over the other control sites, and should be considered as a contaminated not control site. (TIFF) [file pone.0081271.s002.tiff]
